# Bicistronic Gene Transfer Tools for Delivery of miRNAs and Protein Coding Sequences

**DOI:** 10.3390/ijms140918239

**Published:** 2013-09-05

**Authors:** Michelle L. Stoller, Henry C. Chang, Donna M. Fekete

**Affiliations:** 1 Department of Biological Sciences, Purdue University, 915 W State St, West Lafayette, IN 47907-1392, USA; E-Mails: mlstolle@purdue.edu (M.L.S.); hcchang@purdue.edu (H.C.C.); 2 Purdue University Center for Cancer Research, Purdue University, 201 S University Dr, West Lafayette, IN 47907-2064, USA

**Keywords:** miRNAs, gene therapy, miR-183 family

## Abstract

MicroRNAs (miRNAs) are a category of small RNAs that modulate levels of proteins via post-transcriptional inhibition. Currently, a standard strategy to overexpress miRNAs is as mature miRNA duplexes, although this method is cumbersome if multiple miRNAs need to be delivered. Many of these miRNAs are found within introns and processed through the RNA polymerase II pathway. We have designed a vector to exploit this naturally-occurring intronic pathway to deliver the three members of the sensory-specific miR-183 family from an artificial intron. In one version of the vector, the downstream exon encodes the reporter (GFP) while another version encodes a fusion protein created between the transcription factor Atoh1 and the hemaglutinin epitope, to distinguish it from endogenous Atoh1. *In vitro* analysis shows that the miRNAs contained within the artificial intron are processed and bind to their targets with specificity. The genes downstream are successfully translated into protein and identifiable through immunofluorescence. More importantly, Atoh1 is proven functional through *in vitro* assays. These results suggest that this cassette allows expression of miRNAs and proteins simultaneously, which provides the opportunity for joint delivery of specific translational repressors (miRNA) and possibly transcriptional activators (transcription factors). This ability is attractive for future gene therapy use.

## 1. Introduction

MicroRNAs (miRNAs) are small non-coding RNAs that are usually between 22 and 24 nucleotides in length. Each miRNA contains a seed region defined by nucleotides 2–7/8 that is perfectly complementary to a sequence found within its target messenger RNA (mRNA). This bond allows the miRNA to control the levels of its target proteins by downregulating the translation or stability of the target mRNA [1]. Since the discovery of microRNAs (miRNAs), research has focused on identifying conserved miRNA families and determining how these small molecules regulate a multitude of cellular processes that occur during cancer [2,3] and development [4].

In development, a subset of miRNAs has received attention because their expression patterns are relatively specific for distinct cell types or organs. One approach to explore the function of such miRNAs is to either knockdown their levels or to force their overexpression *in vivo* or *in vitro*. Intracellular injection or transfection of miRNA mimics has been successful to overexpress mature miRNAs, although the elevated level of miRNA mimics is transient because they are not stably transduced. As an alternative, exogenous miRNAs can be stably expressed using two distinct transcriptional pathways. Some vectors use the RNA polymerase III (polIII) pathway via the U6 promoter to drive expression of pre-miRNA hairpins [5,6], while others use the RNA polymerase II (polII) pathway, for example to express two pre-miRNA sequences downstream of a tet-responsive PolII promoter [7]. A major drawback of these approaches is that cells overexpressing miRNAs cannot be easily identified, making subsequent phenotypic analysis difficult. To circumvent this problem, it is common to combine the delivery of these miRNA elements with some type of reporter gene using IRES (internal ribosomal entry sites) to make a bicistronic mRNA [2,8] or to deliver miRNAs and a reporter gene using two different promoters. In the latter case, a polII- or polIII-based promoter controls the production of the miRNA and a polII-based promoter drives expression of the reporter gene [9,10]. While the use of two promoters allows production of miRNAs and a protein-coding gene, the production of the two factors is not necessarily coordinated. Such a tenuous link between the relative levels of miRNAs and any associated reporter (such as GFP) could compromise the use of the latter as an estimate of the former in functional studies. An alternative approach to overexpress miRNAs is to generate vectors that resemble the 38% of endogenous miRNA genomic loci where miRNAs are found within the introns of protein-coding genes [11]. When used in this context, both miRNAs and an exogenous gene, such as a *GFP* reporter or cell-surface marker, can be placed under the control of the same polII-dependent promoter [12].

Here we describe the development and functional testing of an intronic cassette to deliver a small family of miRNAs, the miR-183 family, that is specifically expressed in primary sensory cells in variety of vertebrate sensory systems, including vision, hearing, taste, olfaction and somatosensory. The evolutionarily conserved miR-183 family miRNAs has three members (miR-183, -96 and -182) that are transcribed as a single polycistronic pri-miRNA [13]. Although our interest is in the role these miRNAs play in the specification of mechanosensory cells of the inner ear, members of this sensory-specific miRNA family are also upregulated in several different types of cancer [14–16].

*MIR96* was the first miRNA locus to be associated with a hereditary human disease when it was linked to the DFNA50 locus in two families with dominant non-syndromic progressive hearing loss [17]. Each family has a point mutation in the seed region of *MIR96*, but at different nucleotides. A third deafness allele of DFN50 maps to a location in the pre-miR-96 transcript that likely interferes with miRNA processing [18]. Further supporting the link between deafness and mutations in miR-96, a semidominant deaf mouse mutant (diminuendo) was found with yet a third point mutation in the seed region [19]. The physiological and anatomical defects, present in either heterozygous or homozygous diminuendo mice, indicate that hair cells (HC) fail to fully mature [20].

In mouse, *Mir183*, *96* and *182* are located within an intronic region on Chromosome 6, and are transcribed as a single polycistronic pri-miRNA [21,22]. This coordinated expression is restricted to HCs as they begin to differentiate in both mice and zebrafish [23–26], suggesting that these miRNAs participate in HC development. Indeed, morpholino-mediated knockdown of each of the three miRNAs in zebrafish caused smaller inner ear sensory organ size and reduced numbers of HCs 2 days after injection [26]. Furthermore, overexpression of miR-96 and miR-182, by injection of double-stranded miRNA mimics into one-celled zebrafish, generated duplicate inner ears and produced supernumerary and ectopic inner ear HCs [26].

In total, data from humans, mice and zebrafish argue that the miR-183 family is essential for proper HC development and maintenance. As such, they should be considered as potential therapeutic agents for treating deafness due to HC loss. The vast majority (90%) of hearing loss is categorized as sensorineural, of which the most common type results from the destruction or malformation of the HCs occupying the organ of Corti, while sparing the associated supporting cells. One therapeutic approach is to deliver the HC-promoting transcription factor, Atonal1 (Atoh1), to the supporting cells of damaged ears. This has met with some success in animal models [27,28], although further studies are needed. Since it has been established that initiation and maturation of HCs require a complex regulatory network to turn off and on certain genes [29], we reasoned that the reprogramming of supporting cells into HCs might be enhanced by combining the delivery of an activating factor (Atoh1) and repressive elements (the miR-183 family). As every miR-183 family member is present during HC formation, we desired a gene transfer strategy that could efficiently and simultaneously deliver all 3 miRNAs along with a known HC-specification gene (*Atoh1*) to the same target cell population.

We produced two vectors containing the entire miR-183 family within a single artificial intron located upstream of a protein-encoding exon. The exon encoded either GFP as a reporter gene or a traceable version of Atoh1. We demonstrated that this design facilitates the coordinated expression of all three mature miRNAs and the associated protein. The data suggest that by simply exploiting one of the natural miRNA production pathways, it is possible to simultaneously deliver multiple negative and positive regulatory elements. Since many cellular processes require the joint activation and repression of downstream pathways, this delivery system provides an opportunity to achieve that dual manipulation efficiently.

## 2. Results and Discussion

### 2.1. Construction of Bifunctional Atoh1-HA and miRNA Expression Vector

**Figure 1:**
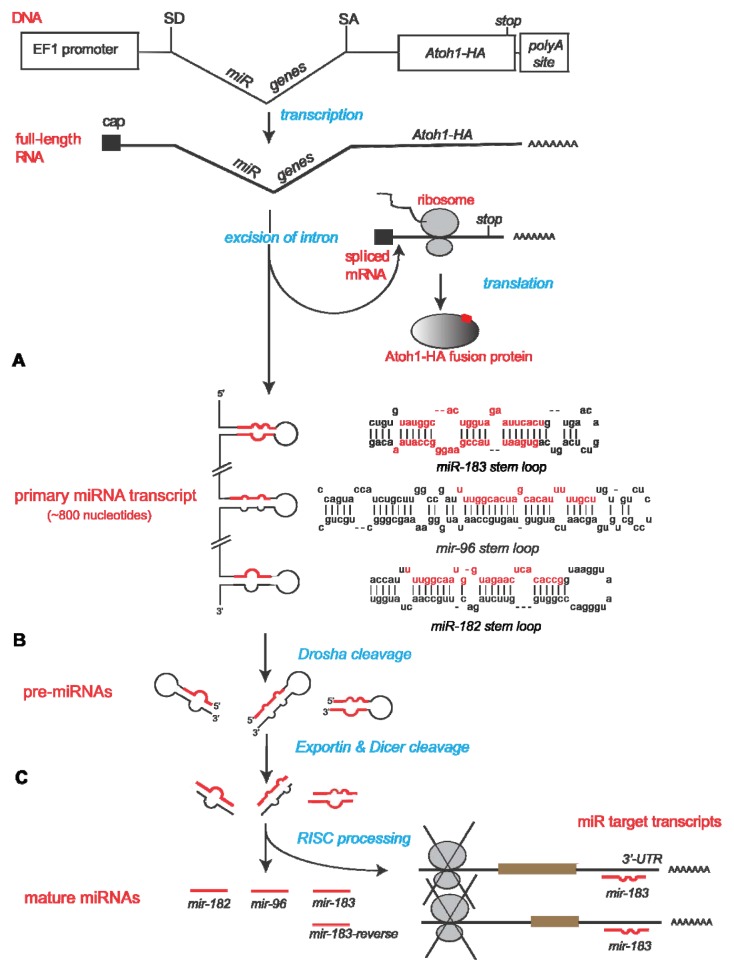
Bifunctional vector design and processing of transcripts. The vector consists of the EF1α promoter that will drive expression of the *miR-183* family of genes from the intron designated by the splice donor (SD) and splice acceptor (SA) site, and an exon encoding Atoh1 fused to the hemagluttinin influenza epitope (HA). Once the plasmid is transcribed into RNA, endogenous enzymes present in transfected cells should recognize the SD and SA sites to release the intron containing the primary miRNA transcript (**A**); clip it into the three distinct pre-miRNAs, export them from the nucleus (**B**); and then further process them into mature miRNAs (**C**). As the miRNAs follow their own maturation pathway, the spliced Atoh1-HA-encoding polyA+ transcript is exported from the nucleus and processed as mRNA.

In order to coordinate the expression of the miRNAs and Atoh1 with high precision within the same cell, both elements are synthesized from the same RNA transcript. To accomplish this, an artificial intron containing the miRNAs is placed downstream of EF1α (human elongation factor 1 alpha; pEF1X [30]) and upstream of *Atoh1* coding sequence (Figure 1). Within the mouse genome, about 3.5 kb of sequence separates *Mir182* from the nearest other family member *Mir96* [21]. To accommodate the size restrictions of certain delivery vectors planned for the future, such as the RCAS avian retrovirus and adeno-associated virus, we removed this large intervening stretch between *Mir182* and *Mir96* while retaining the natural pre-miRNA sequences for all 3 family members. Thus, all of the endogenous sequence between *Mir183* and *Mir96* (~120 bp) along with ~100 bp of sequence flanking the end of each pre-miRNA sequence was kept. Then, the pre-miR-182 sequence, with 120 bps flanking each end, was fused to the *Mir183*/*Mir96* fragment by PCR.

The combined pri-miRNA sequences were inserted into the artificial intron sequence (intron discussed by Lin and colleagues [31,32]). This artificial intron of only ~100 bps contains a splice donor site at the 5′ end of the pri-miRNAs. The 3′ end flanking the pri-miRNAs houses a branch point domain, polypyrimidine tract, and splice acceptor site. The polypyrimidine tract allows spliceosome assembly, while the branch point is necessary for the cell to recognize that a splicing event should occur to excise the element between the splice donor and acceptor sites.

**Figure 2:**
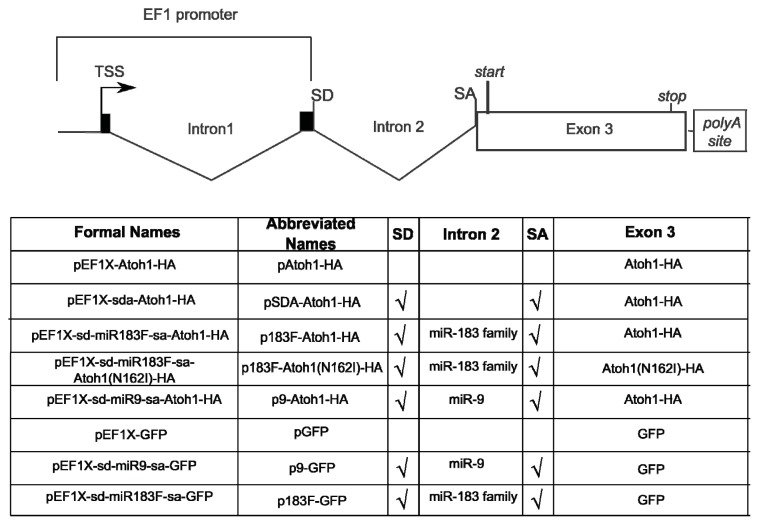
Content and design of overexpression vectors. Each overexpression vector discussed in the paper is listed with its formal name, abbreviated name, and contents. Black boxes represent exons. Intron 1 and exons 1 and 2 were present within the plasmid backbone prior to modification. Checkmarks indicate the presence of artificial intronic flanking sequences. Empty spaces indicate a specific component is not found within that particular vector. TSS: transcription start site.

Downstream of the miRNA intron is the murine *Atoh1* coding region. This *Atoh1* sequence was proven bioactive by its ability to induce ectopic HCs in utero [33]. To facilitate the detection of Atoh1 expression from transfected plasmids, an influenza hemaglutinin (HA) peptide tag (YPYDVPDYA) was fused in-frame to the *Atoh1* coding sequence. Figure 2 displays the overall design of the resulting plasmid, pEF1X-sd-miR183F-sa-Atoh1-HA, hereafter referred to as p183F-Atoh1-HA. Figure 2 also provides details for the introns and exons of the other constructs and their abbreviated names that will be introduced below.

### 2.2. Confirmation of Atoh1-HA Production and Function from a Bifunctional Cassette

**Figure 3:**
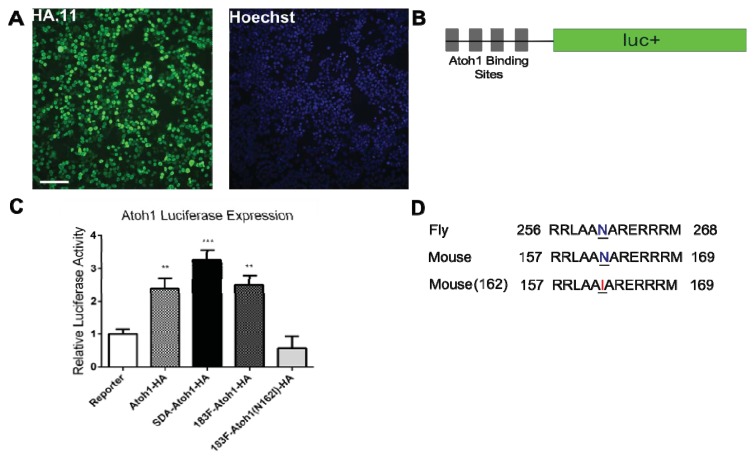
The Atoh1-HA fusion protein is functional and detectable by immunofluorescence. (**A**) Detection of Atoh1-HA fusion protein with HA.11 antibody in cells transfected with p183F-Atoh-HA. Scale bar = 100 microns; (**B**) Illustration of Atoh1 reporter construct; (**C**) Relative luciferase activity of cells transfected with Atoh1 reporter alone or with the indicated versions of the Atoh1-HA overexpression constructs. Luciferase activities are all referenced to cells transfected only with the reporter construct, which is set at 1.0. All constructs showed a significant increase in luminescence compared to the control except p183F-Atoh1(N162I)-HA. Each bar represents mean (±standard error) within each group. Each experiment was replicated at least three times; (**D**) Alignment of conserved Atoh1 segment between fly and mouse. Highlighted is the location of the amino acid mutated to make Atoh1 non-functional while maintaining the HA tag. *p < 0.05, **p < 0.005, ***p < 0.0001.

To ascertain that Atoh1 is expressed from this bicistronic system, HEK293T cells transfected with p183F-Atoh1-HA were stained with anti-HA antibody. In cells 24 h after transfection, HA-positive staining was readily seen in the nuclei using immunofluorescence (Figure 3A), consistent with the fact that Atoh1 is a transcription factor. No HA-positive staining was seen in mock-transfected cells, demonstrating that the signal in p183F-Atoh1-HA-transfected cells is specific (data not shown).

While the immunofluorescence suggests that HA-tagged Atoh1 was expressed and properly localized, it remains possible that the addition of a peptide hinders its bioactivity. To ensure that the HA-tagged Atoh1 is functional, we tested its ability to activate the expression of a luciferase-based reporter gene (4E-box), which has a firefly (FF) luciferase coding sequence placed under the control of four Atoh1-binding sites [34]. In addition, hpRL-SV40 (Promega, Madison, WI, USA), a plasmid with Renilla luciferase driven by a constitutive promoter, was included for normalization. HEK293T cells transfected with pAtoh1-HA showed a 138% increase (*p* = 0.0031) in FF luminescence, compared to those transfected with the pEF1X empty vector. Similarly, cells transfected with pSDA-Atoh1-HA and p183F-Atoh1-HA showed significant increase in FF luciferase luminescence (Figure 3C; pSDA-Atoh1-HA, 225% increase, *p* < 0.0001; p183F-Atoh1-HA, 149% increase, *p* = 0.0004).

To ensure that this increase in FF luciferase expression required a functional Atoh1 protein, we took advantage of the fact that mutating a highly conserved asparagine in the homeobox domain has been shown to disrupt Atoh1 function [35]. We generated p183F-Atoh1(N162I)-HA, which expresses Atoh1-HA with the N162I substitution (this mutation is analogous to the point mutation affecting amino acid 261 in the fly) (Figure 3D). In cells transfected with p183F-Atoh1(N162I)-HA, mutant Atoh1-HA was still detectable by immunofluorescence (data not shown), although its ability to activate FF luciferase expression was diminished (69% decrease in luminescence relative to 3 vectors carrying the wild type *Atoh1* sequence; ANOVA; *p* < 0.0001). Interestingly, the N162I mutation seems to act as a dominant negative, as the luminescence in p183F-Atoh1(N162I)-HA transfected cells decreased by 43% compared to the control (Figure 3C; *p* = 0.8213). Atohl is believed to act as a heterodimer that binds to other bHLH (basic helix loop helix) transcription factors such as E47 [36]. It is likely that expression of N162I prevents the formation of functional Atoh1 heterodimers by depleting the pool of endogenous E47 or other such transcription factors. In any case, our results showed clearly that functional HA-tagged Atoh1 is expressed from these constructs.

### 2.3. Confirmation of miRNA Production and Function from a Bifunctional Cassette

**Figure 4:**
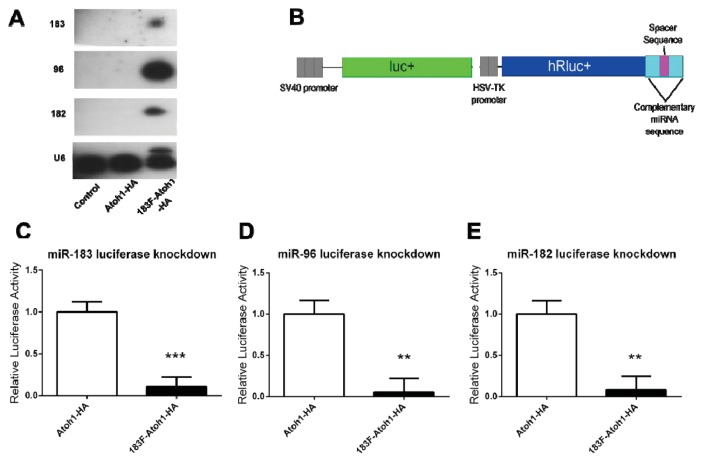
miRNAs are expressed from the 183F-Atoh1-HA vector and functional. (**A**) p183F-Atoh1-HA transfection leads to production of mature miR-183 family members. Untransfected HEK293T cells and cells transfected with pAtoh1-HA show no detectable expression of 183 family members; whereas miR-183, -96, and -182 are each detected in cells transfected with p183F-Atoh1-HA. U6 levels are provided as the loading control; (**B**) Illustration of miRNA-specific reporter plasmids. PsiCHECK-2 luciferase reporters contain 2 sites complementary to miR-96, -182, or -183 in the 3′UTR; (**C**–**E**) Knockdown of luciferase activity in reporters specific to each member of the miR-183 family; (**C**) Cells co-transfected with reporter containing miR-183 sites and p183F-Atoh1-HA showed a marked decrease in luciferase activity compared to wells transfected with the miR-183 reporter and pAtoh1-HA. Experiments in (**D**) and (**E**) were conducted in the same manner except with miR-96 or miR-182 complementary binding sites in the luciferase reporter. All showed significant decrease in luminescence. Each bar represents mean (±standard error) for each group. Each experiment was replicated at least three times. *p < 0.05, **p < 0.005, ***p < 0.0001.

To assess whether the miRNAs were synthesized from the artificial intron, small RNAs collected from HEK293T cells 30 h after p183F-Atoh1-HA transfection were analyzed by Northern blots. While none was detected in untransfected or pAtoh1-HA transfected cells, bands corresponding to mature miRNA of each 183 family member were seen in p183F-Atoh1-HA-transfected cells (Figure 4A). It is notable that the relative levels of the three miRNAs are distinctly different, with miR-96 most prominent. The observation that these family members are not uniformly expressed has also been reported for murine retina [22] and cochlea [24].

A dual luciferase assay system was used to confirm bioactivity of the miR-183 family miRNAs produced from the cassette. For each miRNA, a reporter construct was created beginning with psiCHECK-2 (Promega, Madison, WI, USA), into which two binding sites complementary to a mature miRNA and separated by a spacer sequence were inserted downstream of the *Renilla* luciferase gene (Figure 4B). The psiCHECK-2 vector also contains the firefly luciferase gene driven off a separate promoter, so that luminescence from the firefly protein serves as an internal transfection control. Reporters containing the miRNA binding sites for miR-182, miR-96 or miR-183 were co-transfected withp183F-Atoh1-HA into HEK293T cells. The luminescence ratio (corrected for transfection efficiency) from the experimental wells was compared to control wells transfected with the relevant miR-183 family reporter and the pAtoh1-HA plasmid lacking the miRNA intron. As shown in Figure 4C–E, each miRNA-reporter construct showed a significant knockdown in luminescence compared to its corresponding control (miR-96: 95% knockdown, *p* = 0.0013; miR-182: 92% knockdown, *p* = 0.0008; miR-183: 89% knockdown, *p* < 0.0001). Thus, all 3 miRNAs are produced from the bifunctional cassette and appear functional.

### 2.4. Overexpression of Functional miRNAs from GFP Expression Vectors

**Figure 5:**
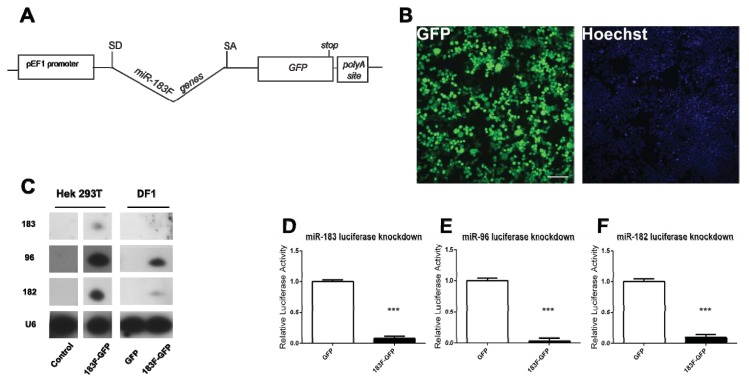
Plasmid containing *miR-183* family and the *GFP* gene produces functional miRNAs and GFP protein in HEK293T cells. (**A**) Vector design of p183F-GFP; (**B**) Visualization of GFP in cells transfected with p183F-GFP. Scale bar = 100 microns; (**C**) The miR-183 family is expressed from p183F-GFP in mammalian and avian cells. Cells transfected with p183F-GFP showed expression of mature miR-183, -96, and -182. Control (untransfected cells) and pGFP transfected cells exhibit no detectable miRNA. U6 levels serve as the loading control; (**D**–**F**) Luciferase activity is decreased by expression of miRNAs from p183F-GFP expressing vector; (**D**) Cells co-transfected with p183F-GFP and the psiCHECK-2 reporter containing sites complementary to miR-183 show a significant decrease in luciferase activity compared to cells co-transfected with the pGFP and reporter; (**E**) and (**F**) show results of experiments similar to (**D)** except the reporter contained different complementary binding sites: 96 for (**E**) and 182 for (**F**). Each bar represents mean (±standard error) for each group. Each experiment was replicated at least three times. *p < 0.05, **p < 0.005, ***p < 0.0001.

While our primary interest involved the dual expression of the miR-183 family and Atoh1, it was also desirable to express the miR-183 family alone to assess how much of an impact this family can have on HC development by themselves. To accomplish this, the *Atoh1-HA* coding region was replaced with *GFP*, which would allow the identification of cells expressing transfected miRNA constructs. Furthermore, as the design of p183F-GFP is the same as the p183F-Atoh1-HA (Figure 5A), phenotypic analysis using vectors with or without *Atoh1-HA* is less likely to be confounded by changes in the processing of the RNA transcripts that may affect transcript levels.

To test whether functional GFP protein is expressed from p183F-GFP, HEK293T cells were transfected and observed not only for direct GFP fluorescence but also after enhancing the signal with anti-GFP antibodies. After 24 h, green emissions were detected from the majority of fixed, transfected cells both before (not shown) and after immunofluorescence (Figure 5B).

All three mature miRNAs of the miR-183 family could be detected in HEK293T cells transfected with p183F-GFP but not in cells transfected with a GFP vector lacking the miRNA intron, as assessed by Northern blots (Figure 5C). Notably, miRNA expression levels appear to remain consistent regardless of the identity of downstream coding sequence (Figure 5C; HEK293T cells).

To ascertain whether avian cells are able to process and express mammalian miRNAs, small RNAs from DF1 cells (chicken embryo fibroblast cells) transfected with p183F-GFP or pGFP were analyzed by Northern blotting. While both p183F-GFP and pGFP transfected groups expressed GFP fluorescence (data not shown), only those transfected with p183F-GFP showed bands corresponding to miR-182, -96 and -183 (Figure 5C; DF1 cells). The relative levels of the miR-183 family members appeared lower in transfected DF-1 cells than HEK293T cells. This discrepancy could result from the species difference of the transfected cells (chicken *vs.* human, respectively) or the difference in their respective tissue origins (embryonic day 10 fibroblasts *versus* fetal kidney, respectively). Nevertheless, our data demonstrated clearly that the miRNAs from p183F-GFP can be processed and produced in avian cells, allowing the option of using them in avian-specific vectors, like the RCAS retroviral vector [37].

Using the miRNA luciferase reporters discussed above, the function of the three miRNAs expressed from p183F-GFP was tested. Compared to HEK293T cells transfected with pGFP (which lacks the miRNA-producing intron), p183F-GFP transfection showed significant decrease in the expression of all three targets (miR-96: 97% knockdown, *p* < 0.0001; miR-182: 91% knockdown, *p* < 0.0001; miR-183: 92% knockdown, *p* < 0.0001) (Figure 5D–F). These observations demonstrated that miRNAs processed from the intron can successfully knockdown miRNA-specific targets.

### 2.5. MiRNAs Produced from Expression Vectors Bind with Specificity

To demonstrate the specificity of the knockdown mediated by these intronic miRNAs, we generated another luciferase-based reporter with two sites complementary to miR-9, a miRNA unrelated to the miR-183 family. Two intronic-miR-9 expression vectors with different downstream protein coding sequences (*Atoh1-HA or GFP*) were also generated to ensure this miR-9 reporter functions properly.

**Figure 6:**
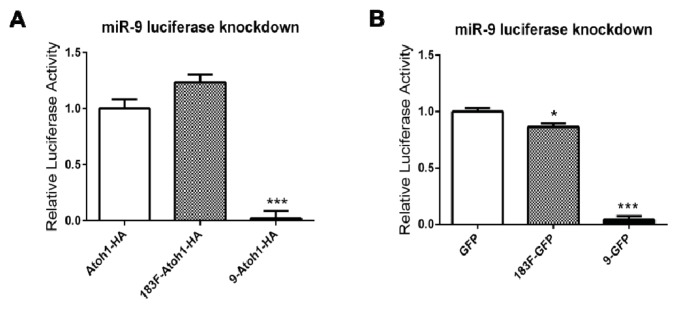
The miRNAs produced from both miRNA expression vectors bind to their targets with specificity. (**A**,**B**) Luciferase activity is decreased when vectors containing miR-9 are co-expressed with the miR-9 luciferase reporter but not when co-expressed with the miR-183 family expressing plasmids; (**A**) Luciferase activity of transfected cells containing the miR-9 luciferase reporter with pAtoh1-HA were compared to cells co-transfected with the miR-9 reporter and p183F-Atoh1-HA or p9-Atoh1-HA; (**B**) Experiments similar to those in A except control cells were co-transfected with miR-9 reporter and pGFP, while experimental cells were co-transfected with reporter and p183F-GFP or p9-GFP. *p < 0.05, **p < 0.005, ***p < 0.0001. Bars represent mean (±standard error) for each group. Each experiment was replicated at least three times.

In cells transfected with miR-9 reporter, co-transfection of p9-Atoh1-HA or p9-GFP resulted in greater than 95% decrease in *Renilla* luciferase expression (98% decrease, *p* < 0.0001 for p9-Atoh1-HA; 96% decrease, *p* < 0.0001 for p9-GFP), demonstrating that these miR-9 expression vectors are functional. The comparable knockdown with both suggests that miR-9 vectors are similar to the 183F-expressing plasmid series in being effectively processed from the artificial intron regardless of the identity of downstream coding sequences. Most importantly, co-transfection of p183F-Atoh1-HA or p183F-GFP, while capable of knocking down the expression of 183F-based reporters (see above), showed only negligible effects on the *Renilla* luciferase level from the miR-9 reporter (23% increase, *p* = 0.10 for p183F-Atoh1-HA; 13% decrease, *p* = 0.02 for p183F-GFP) (Figure 6). These data suggest that intronic miRNAs produced from 183F- and miR-9-expressing vectors regulate the expression of their target genes with high specificity.

## 3. Experimental Section

### 3.1. Bifunctional Plasmid Construction

The murine *Atoh1* coding sequence was PCR amplified from the pEF1-Atoh-IRES-GFP vector, a gift from John Brigande [33]. To facilitate cloning and protein detection, one primer introduced an EcoRI site, while the other primer added an influenza hemaglutinin (HA) tag (YPYDVPDYA) to the *C*-terminus of Atoh1 coding sequence and a *Not*I site (see Table S1). *Atoh1-HA* was cloned into pEF1X (provided by Cliff Ragsdale) [30] as an *EcoR*I–*Not*I fragment and the entire fusion was verified by sequencing (Purdue Genomics Center).

To construct an artificial miR183-containing intron, a *Sal*I–*Hind*III fragment containing a splice donor, three restriction sites *Xba*I, *BamH*I and *Xho*I, polypyrimidine tract, branch point, and a splice acceptor was generated by PCR and cloned into pME-MCS [38], generating pMCS-SDA. PCR primers were generated based on published sequences [31,32]. Then, PCR amplification was used to extract the primary miRNA DNA of all three members of the *miR-183* family from the mouse genome, and to flank the genomic DNA with *Spe*I and *Sal*I. This sequence was then inserted between the *Xba*I and *Xho*I sites found within the intron contained within pME-MCS-sda to create pME-MCS-sd-miR183F-sa. Then, Kpn1 was used to extract the artificial intron containing about 800 bp of mouse *miR-183* family genomic primary miRNA sequence from pME-MCS-sd-miR183F-sa. The *Kpn*I site was used to insert the intron with the *miR-183* family upstream of the Atoh1-HA fusion protein in pEF1X to generate pEF1X-sd-miR183F-sa-Atoh1-HA.

pEF1X-sd-miR183F-sa-GFP vector was constructed by extracting *GFP* from pAAV2.1-CMV-eGFP3-WPRE, provided by Alberto Auricchio [39], via PCR (see Table S3). Using the *Spe*I and *Not*I sites found on the 5′ and 3′ ends respectively, *GFP* was inserted downstream of the miRNA-containing artificial intron in pMCS-sd-miR183F-sa to create pMCS-sd-miR183F-sa-GFP. The pEF1X vector was converted to a Gateway Destination vector (Invitrogen, Carlsbad, CA, USA) by inserting cassette B from the Gateway Conversion Kit (Invitrogen, Carlsbad, CA, USA to create pEF1X-cB. A LR recombination reaction between pMCS-miR183F-GFP and pEF1X-cB generated pEF1X-sd-miR183F-sa-GFP.

pEF1X-sd-miR9-sa-Atoh1-HA and pEF1X-sd-miR9-sa-GFP were constructed in a similar manner as the aforementioned vectors except *Mir9-1* genomic sequence was inserted into the artificial intron instead of the *miR-183* family. PCR was used to extract the endogenous mouse *Mir9-1* sequence and flanking regions using primer sequences described in Shibata *et al.* [9].

For each miRNA reporter, primers were designed to contain two sequences that are fully complementary to the mature miRNA of interest (miR-183, -96, -182, or -9; see Table S4). These sites were separated by a 17 nucleotide spacer sequence. For all cases, the forward primer contains a *Xho*I site, while the reverse primer houses a *Not*I site to allow the resulting PCR fragments to be introduced downstream of the *Renilla* luciferase gene located in the psiCHECK-2 vector (Promega, Madison, WI, USA).

### 3.2. Mutation of Atoh1-HA Fusion Protein

To introduce the N162I substitution in Atoh-1, site-directed mutagenesis was performed with Quikchange 2XL (Agilent Technologies, Santa Clara, CA, USA) according to the manufacturer’s instructions. Primers were designed to induce a point mutation to change the amino acid 162 from Asparagine to Isoleucine in the Atoh1-HA fusion protein encoded by pEF1X-sd-miR183F-sa-Atoh1-HA creating pEF1X-sd-183F-sa-Atoh1(N162I)-HA (see Table S2).

### 3.3. HEK293T Plasmid Transfection

HEK293T were cultured with modified DMEM supplemented with L-glutamine, antibiotics, and 10% calf serum. Using Lipofectamine 2000 (Invitrogen, Carlsbad, CA, USA), cells seeded in 6-well plates were transfected with plasmids of interest. Collection time was assay dependent.

### 3.4. HEK293T Immunostain and Imaging

Cells transfected with pEF1X-sd-miR183F-sa-Atoh1-HA or pEF1X-sd-miR183F-sa-GFP were fixed 24 h post-transfection with 4% paraformaldehyde. The following primary antibodies (1:1000) were used: for detection of the HA tag, anti-HA.11 mouse IgG_1_ monoclonal (Covance, Indianapolis, IN, USA); for detection of GFP, anti-GFP rabbit polyclonal (Molecular Probes, Eugene, OR, USA). Secondary antibodies (1:500) used were Alexa Fluor (Molecular Probes, Eugene, OR, USA) 488 anti-mouse IgG_1_ and Alexa Fluor 488 anti-rabbit IgG. Immunostained cells were imaged under theE800 fluorescence microscope (Nikon, Elgin, IL, USA) with the 20× objective.

### 3.5. Atoh1 and MiRNA Luciferase Assays

The cells, 24 h after transfection, were lysed and luciferase activity was assessed using the dual luciferase assay kit (Promega, Madison, WI, USA) in the Luminoskan Ascent luminometer (Thermo Fisher Scientific, Waltham, MA, USA). For the Atoh1 luciferase assays, the firefly luciferase luminescence readings (at 560nm) were normalized to the *Renilla* luciferase readout (at 480 nm) to account for variation in transfection efficiency. In the case of the miRNA luciferase assays the ratio is inverted: the *Renilla* luciferase readout was normalized to the firefly luciferase readout. These ratios are expressed as relative luciferase activity. Experimental values were referenced to the control values which were arbitrarily set to one. Each treatment condition was conducted at least in duplicate. The experiments were repeated at least three times.

### 3.6. Northern Blots

HEK 293T/17 cells (abbreviated HEK293T cells; ATCC #CRL-11268) or UMNSAH-DF1 cells (abbreviated DF-1 cells; ATTC #CRL-12203) seeded in 35 mm plates were lysed ~30 h post-transfection and small RNAs were collected according to manufacturer’s instructions using the PureLink miRNA Isolation Kit (Invitrogen, Carlsbad, CA, USA). Small RNA (300 ng) was probed for miR-183, -96, or -182 using the High Sensitive miRNA Northern Blot Assay Kit (Signosis, Santa Clara, CA, USA), a chemiluminescence system, according to manufacturer’s instructions.

### 3.7. Statistical Analysis

All results are reported as mean ± standard error. The mean of each group is computed from measurements collected from at least three independent experiments. Statistical significance was determined by using a one-way analysis of variance with block (ANOVA), which was followed by Tukey’s or Tukey-Kramer’s multiple comparisons test (SAS 9.3, SAS Institute, www.sas.com). *p*-values below 0.05 were considered statistically significant.

## 4. Conclusions

Previous research has focused primarily on generating vectors to overexpress siRNAs: small non-coding RNAs that are a perfect complement to their targets. Researchers have shown that it is possible to express multiple siRNAs from one vector using a universal hairpin scaffold within or without an intron under the control of a PolII promoter [40–42]. Others have experimented with siRNA expression designs that allow joint delivery of siRNAs and protein coding regions under control of the same promoter without placement of the siRNA within an intron [43,44]. While these vectors do produce both small RNAs and protein, research shows that it is not the most efficient means of simultaneous delivery due to interference between miRNA/siRNA processing and protein production [45]. Thus, researchers sought to design a dual-delivery vector that would more reliably express a protein-coding gene and siRNA by inserting the siRNA within an artificial intron [31,32,45–47]. Capitalizing on the success of these vectors, others created miRNA vectors with a similar design to deliver one or two miRNAs within an endogenous intron upstream of a cell-surface marker [12]. We took that design one step further and created a dual-delivery vector to express a traceable transcription factor and multiple miRNA genes (in this case an entire miRNA family, using its endogenous sequence, contained within a single artificial intron). Luciferase assays showed that the transcription factor (Atoh1) fused to the HA tag is produced from the vector and is transcriptionally active, while immunofluorescence proved the fusion protein can be detected with HA.11 antibody. With this design, not only can the cells receiving the bifunctional vector be monitored, but artificially-expressed Atoh1 can be distinguished from the endogenous Atoh1 via its tag. Northern blots demonstrated that both human and chicken cells can process the single artificial intron containing 800 bp of endogenous sequence to produce each of the 3 mature miRNAs. These miRNAs were also shown to be functional and bind with specificity to their artificial target sequences.

In order to overexpress just the miRNA family and monitor its expression, we created a vector that replaced *Atoh1-HA* with *GFP*. We opted to keep the miRNAs in an intron upstream (rather than downstream) of a coding exon because research has shown that expressing siRNAs within introns upstream of reporters leads to more stable *GFP* mRNA and better GFP expression [45]. Indeed, GFP expression was robust and efficient: every cell present within the transfected wells was GFP-positive. Northern blots also showed that each intronic miRNA is expressed, while luciferase assays illustrated that these miRNA repress their targets and bind with specificity.

The expression construct reported here uses a polII-based promoter to control the expression of an entire miRNA family within the context of an artificial intron and its downstream *GFP* reporter gene. Naturally co-expressed miRNA family members may function optimally if their overexpression is coordinated with one another for healthy cell function. This same design was used to express a functional transcription factor (in the place of *GFP*) and the miR-183 family, while still maintaining the ability to monitor transfected cells by creating the Atoh1-HA fusion protein. Each cassette was transferred into a Gateway-compatible (Invitrogen, Carlsbad, CA, USA) shuttle vector (unpublished) to facilitate their transfer into alternative delivery vectors such as lentivirus or adenovirus. Such vectors will allow delivery of the cassette into tissues that are difficult to access or to transfect, including into the mouse organ of Corti depleted of HCs. Thus, we believe this novel design can be manipulated for multiple overexpression uses to study a variety of different complex cellular systems and possibly for future therapeutic purposes, including hair cell regeneration.

## Supplementary Information
