# Supplementary material for: Bicistronic Gene Transfer Tools for Delivery of miRNAs and Protein Coding Sequences
Source: Int J Mol Sci. 2013 Sep 5;14(9):18239–55. doi: 10.3390/ijms140918239 (PMC3794778; doi:10.3390/ijms140918239)
Supplement: Supplementary file 1 [file ijms-14-18239-s001.pdf]

Supplementary Information

Table S1. Primer sequences used to construct miR-183 family/Atoh1-HA bifunctional cassette.

Splice donor/acceptor	Sequence	Location
Forward	gcggtcgacgtaatctagaggatccctcgagtactaactggtacctcttc	
Reverse	gcaagcttctgcaggatatcaaaaaaaaaagaagaggtaccagttagtactc	
**miR-183 family**		
183-96 Forward	gcactagtggttgtaggacctccagga	Chr6:30169792-30169810
183-96 Reverse	tccagactatggtccggatcctggctgttcaccagggtagggctg	Chr6:30169333-30169357
182 Forward	cctggtgaacagccaggatccggaccatagtctggaccttgtgtt	Chr6: 30166079-30166102
182 Reverse	gcctcgagcgcccaccctctgccactg	Chr6: 30165809-30165828
**Math1-HA**		
Forward	cgaattcgccaccatgtcccgcctgctgcatgcagaag	
Reverse	cgcgcggccgcctaagcgtaatctggaacatcgtatgggtaactggcctcatcagagtcactgtaatg	

Table S2. Sequences used to introduce Atoh1 mutation.

Atoh1 mutation primers	Sequence
Forward	ggaggctggcagcaatcgcaagggaacgg
Reverse	ccgttcccttgcgattgctgccagcctcc

Table S3. Primers used to amplify GFP from pAAV2.1-CMV-eGFP3-WPRE.

GFP primers	Sequence
Forward	cactagtgccaccatggtgagcaagggcgag
Reverse	gcgcggccgcttacttgtacagctcgtccatgccgagag

Table S4. Primers containing sequences complementary to miRNA of interest for creation of miRNA sensors.

Primers	Sequence
**miR96 sponge**	
Forward	gggctcgagagcaaaaatgtgctagtgccaaacccgggaattcgtt
Reverse	ggggcggccgctttggcactagcacatttttgcttctaggtttaaacg
**miR182 sponge**	
Forward	gggctcgagcggtctgagttctaccattgccaaacccgggaattcgtt
Reverse	ggggcggccgctttggcaatggtagaactcacaccgtctaggtttaaacg
**miR183 sponge**	
Forward	gggctcgagagtgaattctaccagtgccatacccgggaattcgtt
Reverse	ggggcggccgctatggcactggtagaattcacttctaggtttaaacg
**miR9 sponge**	
Forward	gggctcgagtcatacagctagataaccaaagacccgggaattcgtt
Reverse	ggggcggccgctctttggttatctagctgtatgatctaggtttaaacg
